# COVID-19 Associated Central Nervous System Vasculopathy

**DOI:** 10.1017/cjn.2020.109

**Published:** 2020-06-02

**Authors:** Ana R. Matos, Miguel Quintas-Neves, Ana I. Oliveira, Luís Dias, Sofia Marques, Raquel Carvalho, José N. Alves

**Affiliations:** Internal Medicine Department, Hospital de Braga, Braga, Portugal; Neuroradiology Department, Hospital de Braga, Braga, Portugal; School of Medicine, Universidade do Minho, Braga, Portugal; Neurology Department, Hospital de Braga, Braga, Portugal

**Keywords:** COVID-19, SARS-CoV-2, Vasculopathy, Ischemia

A 42-year-old man presented to the emergency department with fever, asthenia, myalgias, dry cough, and hyposmia. After detection of viral nucleic acid from severe acute respiratory syndrome coronavirus 2 (SARS-CoV-2) in a nasopharyngeal swab, the diagnosis of coronavirus disease 2019 (COVID-19) was made. As there were no severe criteria for admittance, the patient was discharged on symptomatic medication.

One week later, he was brought to the hospital due to altered mental status, slowness of movements, and apathy. Neurological examination revealed dysexecutive syndrome, perseveration, and mild dysphonia/dysphagia; there were no pyramidal/extra-pyramidal signs. Blood work-up was unremarkable, and brain computed tomography (CT) showed multiple hypodense lesions involving the white matter, basal ganglia, and thalami. Lumbar puncture revealed mild elevated proteins (0.78 mg/dL) without pleocytosis; cerebrospinal fluid (CSF) viral workup was negative, including SARS-CoV-2. Extensive serological immune and infective panels were negative. Suspecting an inflammatory response related to SARS-CoV-2 infection, intravenous immunoglobulin (30 g/day) was initiated.

**Figure 1:**
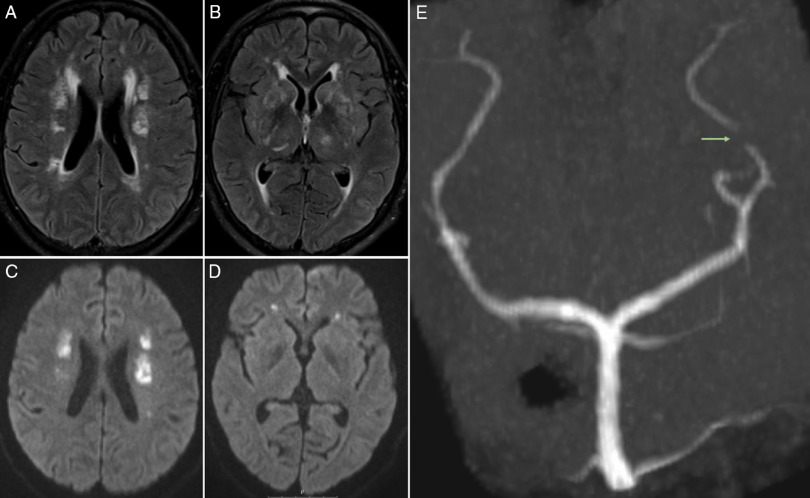
(A, B) Axial fluid-attenuated inversion recovery (FLAIR) sequence depicts multiple hyperintense lesions involving the deep and subcortical white matter on both hemispheres, as well as the thalami and basal ganglia; some of the lesions showed restricted diffusion on diffusion-weighted imaging (DWI) (C, D), namely on both corona radiata and bilateral deep frontal white matter. (E) Three-dimensional time-of-flight (3D TOF) sequence, Maximum intensity projection (MIP) reconstruction on coronal view reveals prominent irregularity (arrow) on the P3 segment of the left posterior cerebral artery.

Brain magnetic resonance imaging (MRI) (Figure 1A–D) confirmed multiple lesions involving the deep and subcortical white matter on both hemispheres, as well as the thalami, basal ganglia, and basal pons, that were hyperintense on fluid-attenuated inversion recovery sequence; some showed restricted diffusion on diffusion-weighted imaging, namely on both corona radiata and bilateral deep frontal white matter. Three-dimensional time of flight (Figure 1E) did not show any major vessel occlusion; however, a prominent irregularity was depicted on the P3 segment of the left posterior cerebral artery. There were no hemorrhages on susceptibility-weighted imaging. Digital subtraction angiography was performed 1 week later, only showing a mild irregularity in a lenticulostriate artery. Cardiac investigation and carotid bulb evaluation by angio-CT were unremarkable. The findings were suggestive of multiple chronologically distinct ischemic lesions on several arterial territories, with the most recent on deep watershed zones; given the current infection by SARS-CoV-2 and presuming an infection-related vasculopathy, immunoglobulin therapy was switched to high-dose intravenous methylprednisolone (1 g/day, 5 days), tapered to 1 mg/kg prednisolone, and aspirin (100 mg/day).

After 13 days, the patient improved and was discharged under corticosteroids, with no new MRI lesions.

Stroke in the setting of viral vasculopathy has been described with other viruses, such as varicella zoster virus (VZV) or cytomegalovirus^1^; more recently, it has also been associated with other coronavirus, namely, Middle East respiratory syndrome coronavirus, during the outbreak in Saudi Arabia in 2012.^2^

The clinical presentation of altered mental status and dysexecutive syndrome, 1 week after being diagnosed with COVID-19, made us consider a probable causal association.

The imaging presentation of multiple lesions involving deep and subcortical white matter, as well as deep gray nuclei, with marked restricted diffusion of some, has been described in the setting of VZV vasculopathy.^3^ In SARS-CoV-2 infection, vascular injury can occur through direct and/or indirect mechanisms: the first as result of viral affinity to angiotensin-converting enzyme-2 expressed by endothelial cells,^4,5^ and the second by misdirected host immune response that induces coagulopathy and vasoconstriction^6^ (possibly without CSF viral isolation). Moreover, the association between SARS-CoV-2 and large vessel occlusion has been recently described.^7^

In our case, vascular imaging did not depict large vessel stenosis/occlusion, only detecting minor irregularities on medium-/small-sized vessels; this could be due to preferential involvement of such arteries or a falsely negative result in the setting of concomitant immunosuppressive therapy. It is also important to note the absence of detectable SARS-CoV-2 ribonucleic acid in CSF could be due to a predominant indirect pathophysiological mechanism of vasculopathy and/or lack of sensitivity of the analytical technique used. Primary angiitis of the central nervous system was also considered, but the absence of obvious large vessel irregularities, normal CSF cellular count, and concomitant SARS-CoV-2 infection led us consider a COVID-19-related vasculopathy as the most probable diagnosis, potentially induced by misdirected immune mediated-vasoconstriction of medium-/small-sized arteries; we believe this represents a new imaging presentation of a SARS-CoV-2-related complication. The patient was managed empirically with steroids and antiplatelets, showing no new lesions on subsequent imaging evaluations.
